# The Permeability of Porous Volcanic Rock Through the Brittle‐Ductile Transition

**DOI:** 10.1029/2022JB024600

**Published:** 2022-06-20

**Authors:** Michael J. Heap, Gabriel G. Meyer, Corentin Noël, Fabian B. Wadsworth, Patrick Baud, Marie E. S. Violay

**Affiliations:** ^1^ CNRS Institut Terre et Environnement de Strasbourg UMR 7063 Université de Strasbourg Strasbourg France; ^2^ Institut Universitaire de France (IUF) Paris France; ^3^ Laboratory of Experimental Rock Mechanics Ecole Polytechnique Fédérale de Lausanne Lausanne Switzerland; ^4^ Dipartimento di Scienze della Terra La Sapienza Università di Roma Rome Italy; ^5^ Earth Sciences Durham University Durham UK

**Keywords:** porosity, permeability, compaction bands, X‐ray computed tomography, volcano deformation, effusive‐explosive transitioning

## Abstract

The permeability of volcanic rock controls the distribution of pore fluids and pore fluid pressure within a volcanic edifice, and is therefore considered to influence eruptive style and volcano deformation. We measured the porosity and permeability of a porous volcanic rock during deformation in the brittle and ductile regimes. In the brittle regime, permeability decreases by a factor of 2–6 up to the peak stress due the closure of narrow pore throats but, following shear fracture formation, remains approximately constant as strain is accommodated by sliding on the fracture. In the ductile regime, permeability continually decreases, by up to an order of magnitude, as a function of strain. Although compaction in the ductile regime is localized, permeability is not reduced substantially due to the tortuous and diffuse nature of the compaction bands, the geometry of which was also influenced by a pore shape preferred orientation. Although the evolution of the permeability of the studied porous volcanic rock in the brittle and ductile regimes is qualitatively similar to that for porous sedimentary rocks, the porosity sensitivity exponent of permeability in the elastic regime is higher than found previously for porous sedimentary rocks. This exponent decreases during shear‐enhanced compaction toward a value theoretically derived for granular media, suggesting that the material is effectively granulating. Indeed, cataclastic pore collapse evolves the microstructure to one that is more granular. Understanding how permeability can evolve in a volcanic edifice will improve the accuracy of models designed to assist volcano monitoring and volcanic hazard mitigation.

## Key Points

- In the brittle regime, permeability decreases by a factor of 2–6, the result of pore throat closure prior to the peak stress
- In the ductile regime, permeability of decreases by an order of magnitude, the result of localized cataclastic pore collapse
- The porosity sensitivity exponent of permeability decreases from elastic to inelastic deformation, indicating granulation

## 1 Introduction

Bubbles form and grow as volatiles dissolved in magma exsolve in response to depressurization during magma ascent in the crust (Coumans et al., 2020; Gardner et al., 1999; Sparks, 1978; Toramaru, 1995). If magma ascent is sufficiently rapid, and if these exsolved volatiles cannot escape the system through permeable networks of bubbles and cracks, the gas pressure within the bubbles can increase (Melnik et al., 2005). In that scenario, magma can fragment if the gas overpressure exceeds a critical threshold value (Koyaguchi et al., 2008; Spieler et al., 2004; Zhang, 1999), a process that is thought to be the origin of explosive eruptive behavior. As a result, the permeability of a volcanic system (the magma and the surrounding host‐rock) is thought to exert influence on eruption style, effusive or explosive. High pore pressures associated with permeability reductions in the volcanic edifice and lava dome are also thought to promote volcanic instability (Ball et al., 2018; Heap et al., 2021; Reid, 2004), and therefore hazards such as the formation of devastating pyroclastic density currents following partial flank collapse (Cole et al., 2015; Glicken, 1986), and erratic explosive behavior (de Moor et al., 2019; Heap et al., 2019; Mick et al., 2021).

A consequence of the importance of the distribution of pore fluids and pore fluid pressure is that many studies have sought to measure and model the permeability of magmas and volcanic rocks, and processes that influence the permeability of these materials have been scrutinized extensively (Blower, 2001; Colombier et al., 2017; Eichelberger et al., 1986; Farquharson et al., 2015; Klug & Cashman, 1996; Kushnir et al., 2017; Mueller et al., 2005; Rust & Cashman, 2004; Saar & Manga, 1999; Vasseur & Wadsworth, 2017; Wadsworth et al., 2021; Wright et al., 2009). Although these studies, and others, have shown that the permeability of volcanic rocks increases as a function of increasing porosity, they have also highlighted that porosity‐permeability relationships for volcanic rocks are complicated by (a) variable genesis (effusive or explosive; e.g., Mueller et al., 2005), (b) variable proportions of pores and cracks comprising their void sspace (e.g., Kushnir et al., 2016), (c) variable pore size and shape (e.g., Blower, 2001; Vasseur & Wadsworth, 2017), (d) variable void space connectivity (e.g., Colombier et al., 2017; Wright et al., 2009), and (e) the variety of additional factors that can influence their porosity structure, such as deformation (Ashwell et al., 2015; Farquharson, Heap, and Baud 2016; Farquharson et al., 2017; Gonnermann et al., 2017; Kennedy et al., 2016) and hydrothermal alteration (Farquharson et al., 2019; Heap, Gravley, et al., 2020; Heap et al., 2021, 2019). Despite these studies, and others, there is still a great deal to learn about the permeability of volcanic rocks, largely due to their variability and microstructural complexity. In particular, few experimental studies have focused on the influence of triaxial deformation (i.e., deformation in the presence of a confining pressure) on the permeability of volcanic rocks in the brittle and ductile regimes.

The mechanical response of a rock—brittle or ductile—to a differential stress depends on the physical properties and microstructural attributes of the rock (e.g., porosity), and the conditions under which it is deformed (e.g., pressure, temperature, and strain rate). Shear fractures develop in porous rock at low pressure and in low‐porosity rock at high and low pressure (i.e., brittle behavior), and delocalized cataclastic flow (grain crushing and pore collapse) or the formation of compaction bands can occur in porous rock at high‐pressure (i.e., ductile behavior) (Baud et al., 2004; Bésuelle et al., 2003; Menéndez et al., 1996; Wong & Baud, 2012). We consider compaction localization as ductile behavior in this contribution (as in the review by Wong & Baud, 2012). Although the mechanical behavior and failure mode of volcanic rocks are complicated by their complex and varied microstructure (Heap & Violay, 2021), experimental studies have also shown that porous volcanic rocks develop localized shear fractures in the brittle regime, and that deformation in the ductile regime can be either distributed or localized (Adelinet et al., 2013; Heap & Violay, 2021; Heap, Baud, et al., 2020; Heap et al., 2015, 2016; Kennedy et al., 2009; Loaiza et al., 2012; Smith et al., 2011; Violay et al., 2015, 2012; Zhu et al., 2011, 2016).

Deformation in both the brittle and ductile regimes is known to influence the permeability of rocks. Throughgoing tensile fractures have been shown to increase the permeability of granite (Nara et al., 2018) and sandstones (Kushnir et al., 2018), shear fractures can increase the permeability of low‐porosity granite and gneiss (Acosta & Violay, 2020; Kluge et al., 2021; Mitchell & Faulkner, 2008) and decrease or unchange the permeability of high‐porosity sandstones (Kluge et al., 2021; Zhu & Wong, 1997), and delocalized cataclastic flow and compaction band formation have been shown to decrease the permeability of porous sedimentary rocks (Baud et al., 2012; David et al., 1994, 2001; Fortin et al., 2005; Meng et al., 2019; Vajdova et al., 2004), with the largest decreases seen for sandstones that develop discrete compaction bands (Baud et al., 2012).

Despite the aforementioned importance, the influence of deformation on permeability is poorly understood for volcanic rocks. Although several studies exist that have shown that through going tensile fractures (“opening‐mode” fractures) increase the permeability of volcanic rock (Eggertsson et al., 2020; Heap & Kennedy, 2016; Heap et al., 2015; Lamur et al., 2017; Nara et al., 2011; Pérez‐Flores et al., 2017; Vasseur & Wadsworth, 2019), fewer studies have performed experiments to understand the influence of a shear fracture on the permeability of volcanic rock. For example, Fortin et al. (2011) measured the permeability of a low‐porosity (0.047 porosity) basalt from Mt Etna volcano (Italy) during deformation at an effective pressure of 20 MPa and found the permeability first decreased from ∼1.65 × 10^−17^ to ∼8 × 10^−18^ m^2^ as differential stress was increased from 0 to ∼300 MPa, before increasing to ∼1.3 × 10^−17^ m^2^ at the point of sample failure (at ∼500 MPa). Following the formation of a macroscopic shear fracture, permeability was measured to be ∼2.9 × 10^−17^ m^2^. Farquharson, Heap, and Baud (2016) measured the permeability of basalt from Mt Etna volcano (0.05 porosity) and andesites from both Volcán de Colima (Mexico; 0.08 porosity) and the Kumamoto Prefecture (Japan; 0.14 porosity) as a function of inelastic strain at an effective pressure of 10 MPa. These authors measured, for all samples, a progressive increase in sample permeability, by up to three orders of magnitude at the maximum imposed inelastic strain, as a function of increasing axial strain.

Similar to the brittle regime, discussed above, few studies have addressed how the permeability of volcanic rock evolves during deformation in the ductile regime. Heap et al. (2015) showed deformed a porous andesite from Volcán de Colima (0.17 porosity) in the ductile regime and found that permeability decreased by about a factor of two at an axial strain of 0.015 and by about an order of magnitude at an axial strain of 0.045. The permeability of a similar andesite from Volcán de Colima (0.26 porosity) decreased by about a factor of three following deformation in the ductile regime to an axial strain of 0.03 (Heap, Baud, et al., 2020). Farquharson et al. (2017) deformed a porous andesite (0.22 porosity), also from Volcán de Colima, at different effective pressures within the ductile regime. These authors found that permeability first increased and then decreased substantially, by up to two orders of magnitude, as a function of increasing inelastic strain. The authors of these studies considered that the measured reductions in permeability were the result of the formation of compaction bands (Farquharson et al., 2017; Heap, Baud, et al., 2020; Heap et al., 2015). However, the aforementioned experimental studies designed to explore the influence of compaction, localized or otherwise, on the permeability of volcanic rocks have been restricted to benchtop measurements performed on samples deformed at high pressure and subsequently unloaded (Farquharson et al., 2017; Heap, Baud, et al., 2020; Heap et al., 2015). Furthermore, our understanding of the influence of compaction localization on the permeability of volcanic rocks is incomplete and warrants further study due to their potential impact on effusive‐explosive transitions, fluid flow compartmentalization, and pore pressure build‐up within a volcanic edifice. This is further justified by the key observation that compaction localization features are known to significantly decrease the permeability of sandstones (Baud et al., 2012; Vajdova et al., 2004), and yet are understudied in volcanic rocks.

Due to the microstructural complexity of porous volcanic rocks, it cannot be assumed a priori that the evolution of the permeability of porous volcanic rocks as a function of strain in the brittle and ductile regimes is similar to that for porous sedimentary rocks. We present here, therefore, an experimental study in which we measured the evolution of porosity and permeability of a porous volcanic rock during triaxial deformation at a range of effective pressures corresponding to both the brittle and ductile regimes.

## 2 Materials and Methods

### 2.1 Experimental Material: Volvic Trachyandesite

**Figure 1:**
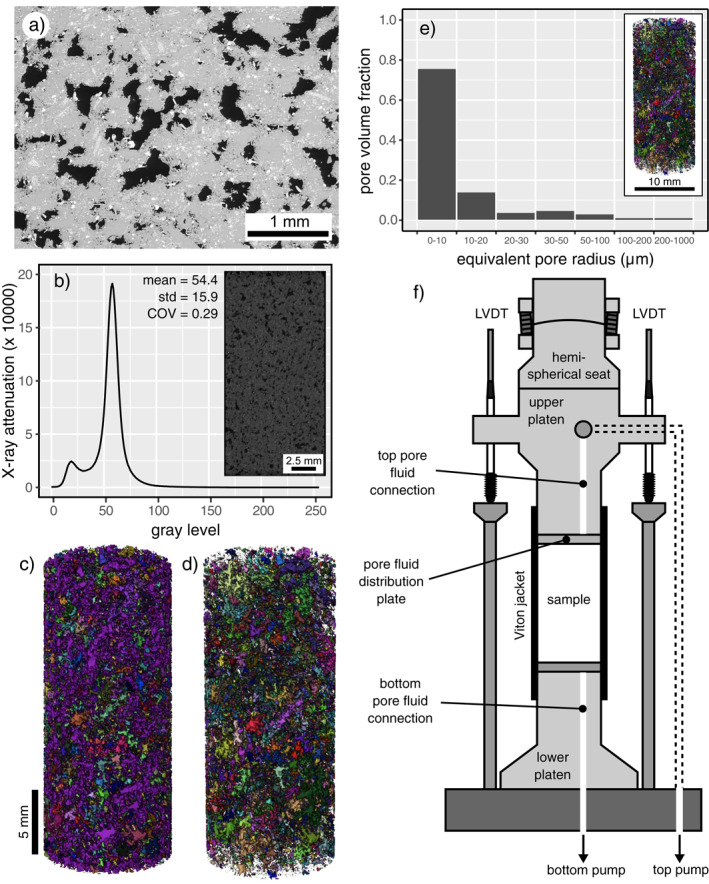
(a) Backscattered scanning electron microscope image of intact Volvic trachyandesite showing the pore structure. Black—void space; gray—groundmass. (b) X‐ray attenuation coefficient as a function of gray level for intact Volvic trachyandesite. A gray level of 0 is black (lowest attenuation) and 255 is white (highest attenuation). The inset shows the measured 2D vertical slice extracted from the 3D X‐ray computed tomography (CT) image volume (cubic voxels of side length 7.17 μm). (c) Segmented 3D X‐ray CT image showing the entire porosity structure of intact Volvic trachyandesite. Each connected pore is shown in a different color (the large purple pore represents the porosity backbone). (d) Segmented 3D X‐ray CT image showing the porosity structure of intact Volvic trachyandesite without the porosity backbone. (e) Pore volume fraction as a function of equivalent pore radius of intact Volvic trachyandesite (for the pores not associated with the porosity backbone). Inset shows an image of the segmented 3D X‐ray CT volume used for the analysis. (f) Schematic of the sample assembly for the triaxial experiments (during the experiments, this sample assembly is within an oil‐filled pressure vessel, not shown; see also Cornelio & Violay, 2020).

A porous trachyandesite collected near the French town of Volvic was selected for this study. The block was sourced from a quarry at Puy de la Nugère, part of the Chaîne des Puys in the French Massif Central. Multiple cylindrical samples were cored to a diameter of 38 mm from the same block and in the same orientation and then precision‐ground (parallelism of the samples was on the order of ±100 μm) to a length of 76–78 mm. These samples were then washed, dried, and vacuum‐saturated with de‐aired, deionized water. Connected porosity, measured using the double weight water saturation method, was measured to be ∼0.21. Total porosity was calculated using the bulk sample density and the solid density determined by measuring the volume of a hand‐powdered sample of known mass using a helium pycnometer (Micromeritics© AccuPyc II). Measurements of total porosity indicated that there is essential no isolated porosity in Volvic trachyandesite. A backscattered scanning electron microscope (SEM) image shows that Volvic trachyandesite consists of irregularly‐shaped pores hosted within a microcrystalline groundmass (Figure 1a). We observe no microcracks within the intact material (Figure 1a).

### 2.2 X‐Ray Computed Tomography (CT)

X‐ray computed tomography (CT) images, with cubic voxels of side length 7.17 μm, of a 10 mm‐diameter sample (∼20 mm in length) were acquired using the high‐resolution X‐ray CT facility at the University of Texas (USA). The inset in Figures 1b and 1a 2D vertical slice extracted from the 3D image volume, shows the features (irregularly‐shaped pores within a groundmass) seen in the SEM image (Figure 1a). The X‐ray attenuation coefficient for this 2D vertical slice is plotted as a function of gray level Figure 1b, where the peaks at gray levels of ∼21 and ∼59 correspond to the pores and groundmass, respectively. The coefficient of variation (COV; the ratio between the standard deviation and the mean X‐ray attenuation), an indication of microstructural heterogeneity, was calculated to be 0.29.

By segmenting the images within the sample using a moment‐preserving bi‐level global segmentation thresholding (Tsai, 1985), we can create 3D images of the porosity structure using image analysis software Avizo©. Figure 1c shows a 3D image of the porosity structure in which each connected pore is shown in a different color. Figure 1c shows that the sample contains a large and very well‐connected porosity backbone, shown in purple. Figure 1d shows the porosity structure of the sample without the porosity backbone (the purple pore shown in Figure 1c), highlighting that there are plenty of pores and porosity clusters (groups of pores) that are unconnected to the porosity backbone (at the resolution of the scan). We find that the pore volumes for the non‐backbone porosity account for 36.2% of the total porosity. Excluding the porosity backbone, the majority of the pores (∼75%) have an equivalent pore radius <10 μm (Figure 1e).

### 2.3 Deformation Experiments and Permeability Measurements

Deformation experiments were performed using the Fluid Induced eaRthquake Simulator (FIRST; Cornelio & Violay, 2020; Noël et al., 2021) triaxial apparatus at the Laboratory of Experimental Rock Mechanics (Ecole Polytechnique Fédérale de Lausanne, Lausanne, Switzerland) (Figure 1f). The permeability of cylindrical samples was measured during constant strain rate deformation under different effective pressures *P*_e_, assumed here to be *P*_e_ = *P*_c_ − *P*_p_, where *P*_c_ is the confining pressure, and *P*_p_ is the pore fluid pressure. We highlight that we only measured permeability in one direction in this study, parallel to the axis of the cylindrical sample (i.e., parallel to the maximum principal stress) (Figure 1f); for simplicity we refer to this measure of sample permeability as “permeability” in this contribution.

Water‐saturated (de‐aired, deionized water) samples were inserted in a Viton© jacket, sandwiched between two 5 mm‐thick pore fluid distribution plates (a schematic of the sample assembly is shown in Figure 1f), and then placed inside a pressure vessel rated to 200 MPa. The endcaps either side of the sample were connected to independent servo‐controlled pore fluid pressure pumps equipped with encoders. The confining pressure (applied using oil), controlled by a pair of servo‐controlled pumps, and the pore fluid (using de‐aired, deionized water) pressure were first increased to 12 and 10 MPa, respectively. Once the samples equilibrated to these pressures, the samples were taken to their target effective pressure by increasing the confining pressure (pore fluid pressure was maintained at 10 MPa using the pore fluid servo‐controlled pump). Experiments were performed at effective pressures of 20, 40, 60, 90, 120, and 150 MPa. The effective pressure required for the brittle‐ductile transition was previously determined to be in the window 60–80 MPa for this material (Heap & Violay, 2021), and so our chosen pressure range spans the brittle‐ductile transition (three experiments in the brittle regime and three in the ductile regime).

Following sample equilibration to the target effective pressure, the permeability of the sample was first measured under hydrostatic conditions (i.e., the differential stress, *Q*, was zero; *Q* = *σ*_1_ − *P*_c_, where *σ*_1_ is the axial stress). To do so, a pore pressure difference of 0.2 MPa was set between the pore fluid pressure pumps upstream and downstream of the sample (i.e., the pumps were set at 9.9 MPa downstream of the sample and 10.1 MPa upstream of the sample). The movement of water through the sample, tracked by the encoders, provided the volumetric flow rate required to calculate permeability using Darcy’s law. We assumed steady‐state flow once the displacement of the upstream and downstream pore fluid pumps was constant. Although steady‐state flow was typically achieved in several minutes, we waited several tens of minutes to be sure that the flow rate was indeed constant. Once steady‐state flow had been established, the pore pressure was set back to a constant 10 MPa applied via one of the pore fluid pressure pumps, and the other pore fluid pressure pump was isolated from the sample. Isolating one of the pore fluid pressure pumps during deformation allowed for the accurate determination of pore volume change, converted to porosity change using the bulk sample volume, as a function of axial strain. The sample was then deformed at a constant displacement rate, corresponding to a constant strain rate of 10^−5^ s^−1^, using an external linear variable differential transducer located on the top of the piston. Axial strain was calculated using the average of two internal linear variable differential transducers and the initial sample length. A hemispherical seat was used to ensure there was no misalignment during loading. Axial force, calculated using the pressure acting on the piston, was converted to axial stress using the sample radius. The permeability of the sample was measured intermittently during the deformation. To do so, loading was first halted, a pressure differential of 0.2 MPa was then set between the pore fluid pressure pumps. Once steady‐state flow had been established, permeability was calculated from the resultant volumetric flow rate using Darcy’s law. This process was continued up to an axial strain of 0.08–0.12. This method of measuring permeability during deformation has been used previously on porous sandstones (Baud et al., 2012; Fortin et al., 2005; Zhu & Wong, 1997) and porous limestones (Meng et al., 2019), allowing our new data for volcanic rocks to be easily compared with previously published data. We adopt the convention that compressive stresses and strains are positive.

We performed an additional experiment in which we measured the porosity change and permeability of a sample during hydrostatic pressurization up to a *P*_e_ of 150 MPa, using the same method described above.

## 3 Results

### 3.1 Porosity and Permeability During Hydrostatic Loading

**Figure 2:**
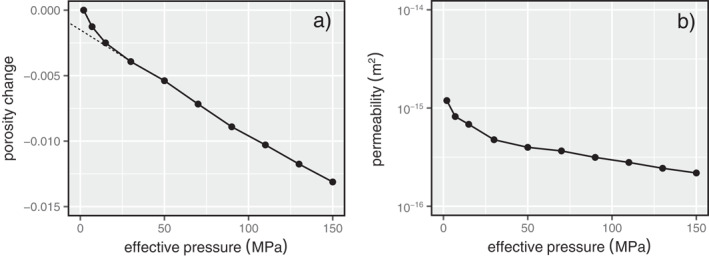
Porosity change (a) and permeability (b) as a function of effective pressure for Volvic trachyandesite during hydrostatic loading. The dashed line in panel (a) is a linear fit to the linear portion of the data set, for which the *y*‐intercept is interpreted to represent the microcrack porosity (∼0.001) (Walsh, 1965). The porosity change shown in panel (a) is the change in sample porosity from an effective pressure of 2 MPa (porosity change at *P*_e_ = 2 MPa is therefore zero).

The porosity and permeability of Volvic trachyandesite both decrease nonlinearly as a function of *P*_e_ (Figure 2; data are provided in a Microsoft Excel© spreadsheet that accompanies this contribution as Supplementary Material). Decreases in porosity and permeability are high as *P*_e_ is increased from 2 to 20 MPa (Figure 2). Porosity and permeability continue to decrease up to the maximum *P*_e_ of 150 MPa, but the decrease in porosity and permeability per unit pressure is lower than that between 2 and 20 MPa (Figure 2). Over the entire pressure range tested, the porosity is reduced by ∼0.013 and permeability is reduced by about an order of magnitude, from 1.19 × 10^−15^ to 2.18 × 10^−16^ m^2^ (Figure 2). Microcrack porosity, estimated using the porosity change data (see Walsh, 1965), is ∼0.001 (Figure 2a) and therefore forms a very small fraction of the total porosity of the sample (∼0.21).

### 3.2 Triaxial Mechanical Data

**Figure 3:**
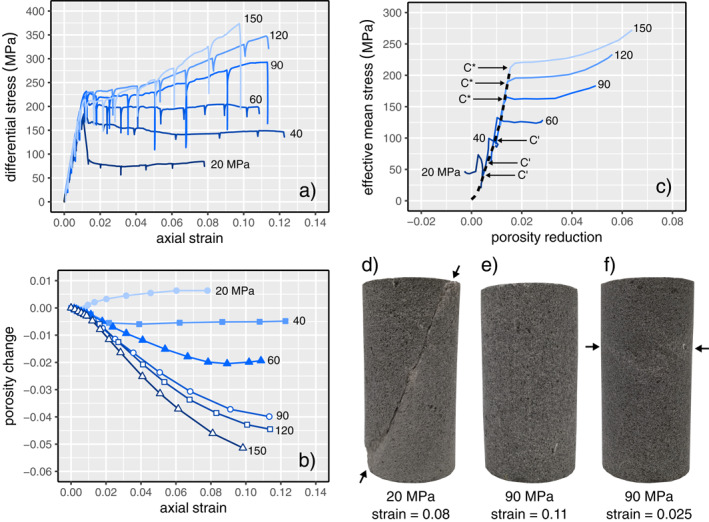
Stress‐strain curves (a) and porosity change as a function of axial strain (b) for Volvic trachyandesite deformed under different effective pressures (indicated by the number next to each curve). Black symbols—brittle experiments; white symbols—ductile experiments. The stress drops in the stress‐strain curves is the result of stress relaxation as the position of the piston is fixed during the measurement of permeability. (c) Effective mean stress as a function of porosity reduction for Volvic trachyandesite deformed under different effective pressures (indicated by the number next to each curve). The hydrostatic curve is shown as a dashed line. The approximate positions of the onset of dilatancy (*C*′) and the onset of inelastic compaction (*C* ^∗^) are labeled for the brittle and ductile experiments, respectively. (d–f) Photographs of samples deformed at (d) an effective pressure of 20 MPa to an axial strain of ∼0.08 showing a shear fracture (indicated by arrows), (e) an effective pressure of 90 MPa to an axial strain of ∼0.11, and (f) an effective pressure of 90 MPa to an axial strain of ∼0.025 showing compaction localization (indicated by arrows). Note the absence of shear fractures at effective pressure of 90 MPa.

For the experiments presented here at effective pressure 20 ≤ *P*_e_ ≤ 150 MPa, the evolution of differential stress and porosity change with increasing axial strain are shown in Figures 3a and 3b, respectively. Effective mean stress, *P*, defined as *P* = (*σ*_1_ + *σ*_2_ + *σ*_3_)/3 − *P*_p_ where *σ*_1_, *σ*_2_, *σ*_3_ are the maximum, intermediate, and minimum principal stresses, is plotted as a function of porosity reduction in Figure 3c. All the data are provided in a Microsoft Excel© spreadsheet that accompanies this contribution as Supplementary Material.

The three experiments performed at constant 20 ≤ *P*_e_ ≤ 60 MPa can be classified as brittle: their mechanical data show evidence of strain softening (i.e., a stress drop, the magnitude of which is reduced at higher *P*_e_) following a peak differential stress (Figure 3a), and all three samples contained a macroscopic shear fracture, ∼30° to the maximum principal stress, when retrieved from the jacket at the end of the experiment (Figure 3d). Following the stress drop, the differential stress remained more‐or‐less constant as a function of increasing axial strain (Figure 3a). This residual differential stress represents the frictional sliding stress, whereat the strain is accommodated by the sliding of the newly‐formed shear fracture. Only the porosity of the sample deformed at 20 MPa increased as a function of increasing axial strain, following a small decrease up to an axial strain of ∼0.05 (Figure 3b). For the sample deformed at 20 MPa, the porosity increase resulting from inelastic deformation can be clearly seen in Figure 3c as the deviation from elastic compaction defined by the hydrostatic curve (the dashed curve on Figure 3c). This marks the position of the onset of dilatational microcracking, termed *C*′ (as labeled on Figure 3c). At 40 MPa, the porosity decreased up to an axial strain of ∼0.02, but remained essentially constant up to an axial strain of ∼0.12 (Figure 3b). The porosity of the sample deformed at 60 MPa decreased up to an axial strain of ∼0.09, and then increased slightly as axial strain was increased from ∼0.09 to ∼0.11 (Figure 3b). We find that the stress required for *C*′ increases as a function of *P*_e_ (Figure 3c).

The three experiments performed at 90 ≤ *P*_e_ ≤ 150 MPa can be classified as ductile: their mechanical data show evidence of strain hardening rather than strain softening characterized by an increase in differential stress with increasing strain (Figure 3a). None of these samples contained a macroscopic shear fracture at the end of the experiment (compare Figure 3d with Figure 3c). Instead, the samples were barreled (Figure 3e). An additional sample deformed to an axial strain of ∼0.025 at an effective pressure of 90 MPa showed evidence of compaction localization (i.e., compaction bands; Figure 3f). The porosity of the samples deformed at effective pressures in the range 90–150 MPa decreased as a function of increasing axial strain, and the porosity decrease per unit axial strain was higher for experiments performed at higher effective pressures (e.g., 150 MPa) compared with those at lower effective pressures (e.g., 90 MPa; Figure 3b). Porosity loss at the end of the experiments was ∼0.04, ∼0.045, and ∼0.05 for the experiments performed at *P*_e_ of 90, 120, and 150 MPa, respectively (Figure 3b). The deviation from elastic compaction defined by the hydrostatic can be clearly seen in Figure 3c, marking the position of the onset of shear‐enhanced compaction (termed *C* ^∗^). We find that the stress required for *C* ^∗^ increases as a function of *P*_e_ (Figure 3c).

### 3.3 Permeability During Nonhydrostatic Loading

**Figure 4:**
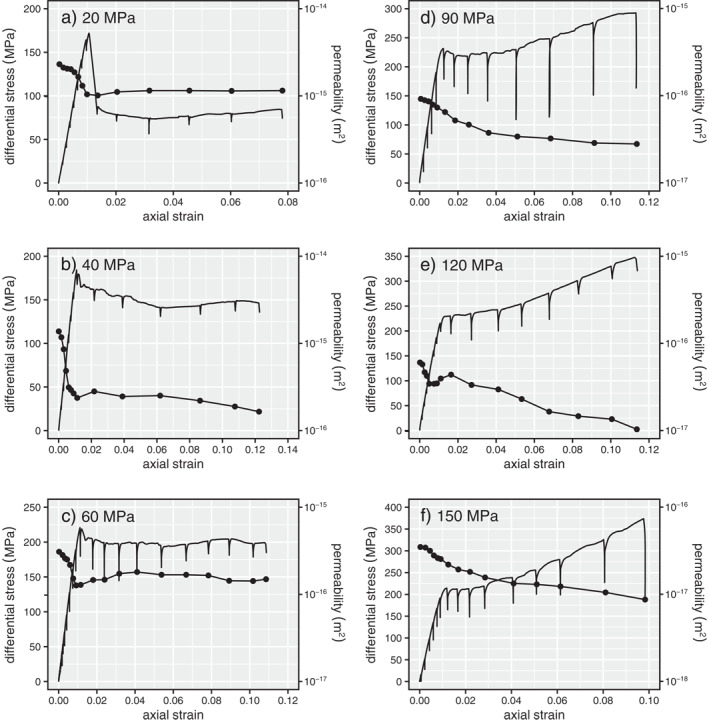
Stress‐strain curves and the evolution of permeability as a function of axial strain for Volvic trachyandesite deformed at effective pressures of (a) 20 MPa, (b) 40 MPa, (c) 60 MPa, (d) 90 MPa, (e) 120 MPa, and (f) 150 MPa.

Figure 4 shows the stress‐strain curves for each of the six triaxial experiments (effective pressures of 20 ≤ *P*_e_ ≤ 150 MPa) repeated from Figure 3a, but here shown together with the data for the permeability measured at steps of increasing axial strain (data are provided in a Microsoft Excel© spreadsheet that accompanies this contribution as Supplementary Material). As before, we can separate these into the three brittle experiments (20 ≤ *P*_e_ ≤ 60 MPa) and the three ductile experiments (90 ≤ *P*_e_ ≤ 150 MPa). For the three brittle experiments, permeability first decreased, by a factor of 2–6, as the sample was loaded to the peak stress (Figures 4a–4c). Following the peak stress, there was a small increase in permeability, followed by either little to no change in permeability (for the experiments at 20 and 60 MPa) or a further decrease in permeability (for the experiment at 40 MPa) (Figures 4a–4c). The permeability of the samples deformed in the ductile regime (effective pressures of 90 ≤ *P*_e_ ≤ 150 MPa) also decreased during the initial loading of the sample (Figures 4d–4f). After the initial phase, permeability then decreased further as a function of increasing axial strain (Figures 4d–4f), although we note that there was a small increase in permeability prior to strain hardening in the experiment performed at 120 MPa (Figure 4e). Reductions in permeability in the ductile regime were up to an order of magnitude (Figures 4d–4f).

**Figure 5:**
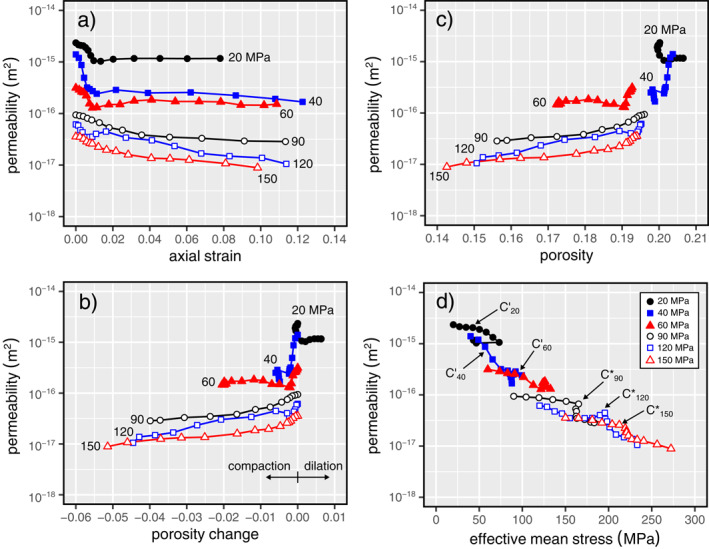
Permeability as a function of axial strain (a), porosity change (b), porosity (c), and effective mean stress (d) for Volvic trachyandesite deformed at effective pressures of 20 ≤ *P*_e_ ≤ 150 MPa (indicated by the number next to each curve). The approximate positions of the onset of dilatancy (*C*′) and the onset of inelastic compaction (*C* ^∗^) are labeled in panel (d) for the brittle and ductile experiments, respectively.

The permeability data are plotted together in Figure 5, which shows permeability as a function of strain for all samples (Figure 5a), and as a function of porosity change (Figure 5b) and absolute porosity (Figure 5c) measured from the start of nonhydrostatic loading. Finally, Figure 5d shows permeability as a function of the effective mean stress, *P*. Hydrostatic and nonhydrostatic loading is coupled in a conventional triaxial experiment. Because the confining pressure is maintained constant, if the axial stress increases by an increment $\Delta \sigma_{1}$ then the differential stress and the mean stress will increase by $\Delta \sigma_{1}$ and $\Delta \sigma_{1}/3$, respectively. Therefore, for a poroelastic material, the volumetric strain (i.e., porosity change) should be controlled solely by the mean stress (Wong et al., 1997). It is for this reason why it is interesting to plot permeability as a function of *P*. The onset of inelastic damage in the brittle and ductile regimes (i.e., *C*′ and *C* ^∗^, respectively) are also labeled on Figure 5d.

The data of Figures 5a–5c show that (a) the initial permeability of the sample (i.e., *Q* = 0 MPa) is lower at higher effective pressures and (b) that, regardless of whether the sample was brittle or ductile, or whether the sample experienced net compaction or net dilation, the final permeability of the sample was always lower than the initial permeability. Figure 5d shows the absolute rate of change of permeability as a function of effective mean stress, |d*k*/d*P*|, increases when *P* > *C*′ in the brittle regime and when *P* > *C* ^∗^ in the ductile regime. The experiment performed at 120 MPa, however, was associated with an increase in the rate of change, followed by a decrease, at *P* > *C* ^∗^ (Figure 5d).

## 4 Discussion

### 4.1 Porosity and Permeability Evolution During Hydrostatic Loading

Our hydrostatic data show that porosity and permeability decrease as a function of *P*_e_ (Figure 2), as observed previously for volcanic rocks (Fortin et al., 2011; Heap et al., 2018; Nara et al., 2011; Vinciguerra et al., 2005). In previous studies on volcanic rocks, large reductions in permeability as *P*_e_ was increased were considered the consequence of the closure of pre‐existing microcracks, often abundant in volcanic rocks. Fortin et al. (2011), for example, measured a permeability reduction of about three orders of magnitude in Etna basalt, a rock with a pre‐existing microcrack network, as *P*_e_ was increased to 150 MPa. For the same increase in *P*_e_, the permeability of Volvic trachyandesite was only reduced by an order of magnitude (Figure 2b). Volvic trachyandesite, however, does not contain obvious microcracks (Figure 1a) and the porosity change data during hydrostatic loading suggest a very low microcrack porosity (Figure 2a). In the absence of abundant microcracks, we consider that the permeability reduction in Volvic trachyandesite at *P*_e_ > 20 MPa are the result of the closure or “pinching” of small‐diameter pore throats that help connect the porosity backbone (shown in purple in Figure 1c). Based on our equivalent pore analysis (Figure 1e) and our microstructural observations using the SEM (e.g., Figure 1a), we consider that these small‐diameter pore throats are on the order of a couple of tens of microns in diameter.

### 4.2 Permeability Evolution in the Brittle Regime

Our data show that in the brittle regime, both increases (as seen at 20 MPa) and decreases (40 and 60 MPa) to sample porosity are associated with a decrease in permeability (Figures 4 and 5). This phenomenon has been observed for porous sandstones (Baud et al., 2012; Zhu & Wong, 1996, 1997) and porous limestones (Meng et al., 2019).

The majority of the measured permeability decrease in Volvic trachyandesite occurred during loading to the peak stress (Figure 4). We therefore conclude that nonhydrostatic loading resulted in the closure or “pinching” of additional pore throats at higher stresses and/or that microcracking surrounding larger pores resulted in the blockage of pore throats, and that the influence of the closure or blockage of these small‐diameter pore throats on the permeability of the sample must have outweighed the influence of the formation and growth of new microcracks following *C*′, even at stresses very close to the peak stress. Indeed, the porosity of the samples deforming in the brittle regime decreased up to the peak stress (Figure 4b). These data are supported by recent image correlation analyses for volcanic rock deforming in the brittle regime, which showed evidence for the compaction of void space in off‐fault volumes of the sample prior to the formation of the macroscopic shear fracture (McBeck et al., 2019; see also; Heap, Baud, et al., 2020; see also image correlation analysis for porous sedimentary rocks in the brittle regime by; Kandula et al., 2022).

Following the formation of a macroscopic shear fracture, the porosity of the samples deformed at 20 and 40–60 MPa increased and decreased, respectively (Figure 3b). However, the permeability remained more‐or‐less constant (Figure 4). The macroscopic shear fracture and microcracks that formed adjacent to the shear fracture likely increased the local porosity (supported by the aforementioned image correlation analyses; Heap, Baud, et al., 2020; McBeck et al., 2019) and therefore connectivity. However, the formed shear fractures did not connect the top and bottom of the samples (e.g., Figure 3d) and so the pore fluid was required to flow through volumes of rock largely unaffected by the deformation (due to the strain localization; Figure 3d), potentially explaining why the permeability of the sample did not increase significantly following shear fracture formation. We also note that, using a network model consisting of tubes and cracks, Zhu and Wong (1996) concluded that microcracks which dilate the void space may also increase the flow path tortuosity, leading to a reduction in sample permeability. Although data from some studies suggest that the permeability of a fracture could be reduced as a function of sliding on the fracture (e.g., Crawford et al., 2008; Morrow et al., 1984; Zhang et al., 1999), we cannot conclude as such with these data (again, because the shear fractures did not connect the top and bottom of the samples, and are inclined with respect to the axis of the cylindrical samples). However, we can conclude, due to the more‐or‐less constant sample permeability during frictional sliding (Figure 4), that the permeability of the fracture was not reduced to below that of the host‐rock. Because the permeability of the sample can be considered as a series (host‐rock, fault, host‐rock), the permeability of the sample would decrease if the permeability of the fault decreased, even if the permeability of the host‐rock increased as a result of dilatant microcracking.

**Figure 6:**
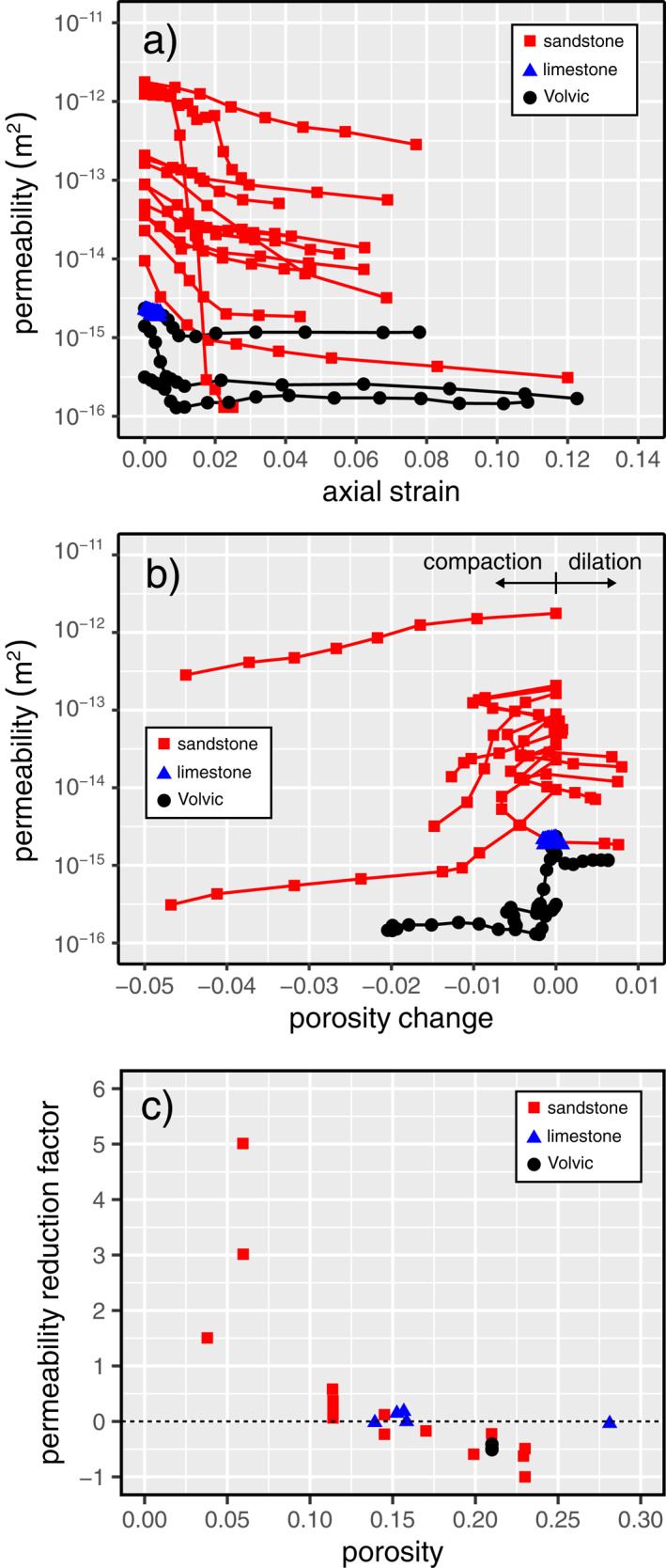
Permeability as a function of axial strain (a) and porosity change (b) for Volvic trachyandesite (black circles) and compiled data for sandstones (red squares; data from Baud et al., 2012; Zhu & Wong, 1997) and limestones (blue triangles; data from Meng et al., 2019) in the brittle regime. (c) Permeability reduction factor, *ξ*, (see Equation 1) as a function of porosity for Volvic trachyandesite (porosity of ∼0.21; black circles) and compiled data for sandstones (red squares; data from Baud et al., 2012; Gatto, 1984; Keaney et al., 1998; Mordecai & Morris, 1971; Senseny et al., 1983; Wong & Zhu, 1999; Zhu & Wong, 1997) and limestones (blue triangles; data from Meng et al., 2019).

We compare the evolution of the permeability of Volvic trachyandesite in the brittle regime with published data for porous sandstones and limestones in Figure 6. Figures 6a and 6b show permeability as a function of axial strain and porosity change, respectively. Data for porous sandstones and limestones are in broad agreement with our new data for volcanic rock: permeability always decreases as a function of increasing axial strain (Figure 6a), regardless of whether the bulk porosity of the sample increased or decreased (Figure 6b).

The evolution of permeability prior to the formation of a macroscopic shear fracture, but following the onset of dilatational microcracking (*C*′), in the brittle regime can be evaluated using “permeability reduction factor”, *ξ* (Wong & Zhu, 1999; Zhu & Wong, 1997):

(1) $$\xi =\;\frac{k_{\text{peak}}}{k_{C^{\prime }}}-1,\;$$

where *k*_peak_ and $k_{C^{\prime }}$ are the permeabilities of the sample at the peak stress and at *C*′, respectively. We plot *ξ* for Volvic trachyandesite deformed at 20, 40, and 60 MPa alongside compiled data from the literature (for sandstones and limestones) as a function of initial porosity in Figure 6c. The permeability of samples with *ξ* > 0 increases as the rock approaches macroscopic failure, and samples characterized by *ξ* < 0 show decreases in permeability approaching failure. Our data for porous volcanic rock, which all show *ξ* < 0, are in agreement with the proposed porosity threshold of 0.15 for permeability‐increasing and permeability‐decreasing behavior in the brittle regime (Zhu & Wong, 1997, Figure 6c). Below this porosity threshold, the samples have a low initial permeability and stand to benefit, in terms of their permeability, from the presence a shear fracture; however, the microstructure and fluid flow paths in high‐porosity rocks, that are already high‐permeability, can be disrupted by the presence of a shear fracture, resulting in a decrease in permeability. Although *k*_peak_ and $k_{C^{\prime }}$ were not measured for the low‐porosity volcanic rocks (0.05–0.15 porosity) deformed by Farquharson, Heap, and Baud (2016), these authors found that brittle deformation was associated with permeability increases, in agreement with the aforementioned porosity threshold.

### 4.3 Permeability Evolution in the Ductile Regime

**Figure 7:**
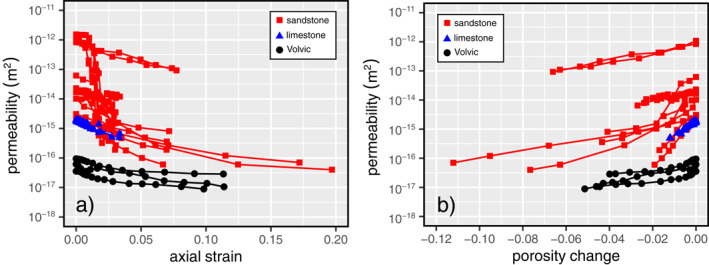
Permeability as a function of axial strain (a) and porosity change (b) for Volvic trachyandesite (black circles) and compiled data for sandstones (red squares; data from Baud et al., 2012; Fortin et al., 2005; Zhu & Wong, 1997) and limestones (blue triangles; data from Meng et al., 2019) in the ductile regime.

In the ductile regime, our data show that both porosity and permeability decrease as a function of increasing axial strain (Figures 4 and 5), as also observed for porous sandstones (Baud et al., 2012; Fortin et al., 2005; Zhu & Wong, 1996, 1997) and porous limestones (Meng et al., 2019). We compare the evolution of the permeability of Volvic trachyandesite in the ductile regime with published data for porous sandstones and limestones in Figure 7. Our new data for volcanic rock are in broad agreement with the data for porous sandstones and limestones: porosity and permeability always decrease as a function of increasing axial strain (Figure 7).

**Figure 8:**
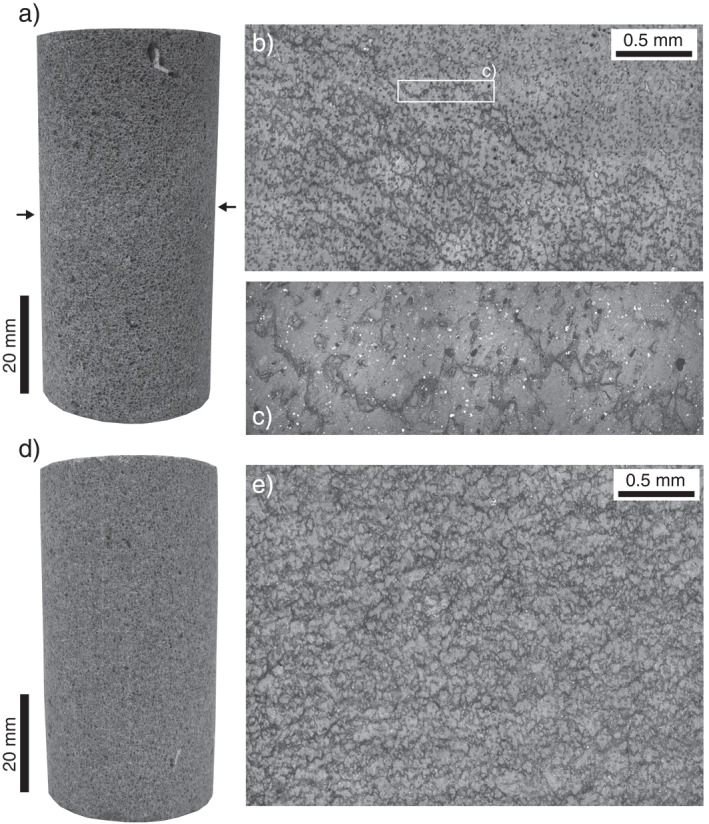
(a) Photograph of a sample Volvic trachyandesite deformed at 90 MPa to an axial strain of ∼0.025 containing a cluster of compaction bands (indicated by the black arrows). (b) Optical microscope image of the sample shown in panel (a) showing the compaction bands, taken in reflected light. (c) Zoomed‐in optical microscope image showing an individual compaction band. (d) Photograph of a sample Volvic trachyandesite deformed at 90 MPa to an axial strain of ∼0.11. (e) Optical microscope image of the sample shown in panel (d) showing “granulation” of the microstructure at large strains, taken in reflected light.

Reductions in porosity, and therefore void space connectivity and permeability, are the result of cataclastic pore collapse in the ductile regime (Zhu et al., 2010), as shown in the optical microscope images provided in Figures 8b and 8c (compare these with Figure 1a). Cataclastic pore collapse has been previously observed during the deformation of porous volcanic rocks in the ductile regime (Adelinet et al., 2013; Heap & Violay, 2021; Heap, Baud, et al., 2020; Heap et al., 2015, 2016; Loaiza et al., 2012; Zhu et al., 2011, 2016). Similar to other porous lavas (see review in Heap & Violay, 2021), the compaction in our Volvic trachyandesite samples localized into compaction bands (Figures 2e and 8a–8c). The compaction bands that formed in the Volvic trachyandesite represent planes of collapsed pores (Figures 8a–8c). We found that many bands grew at the same position along the length of the sample (Figures 8a and 8b), and that the individual bands were a couple of hundred microns in thickness (i.e., approximately the diameter of a pore) (Figure 8c). Compaction bands typically form sub‐perpendicular to the maximum principal stress (see reviews by Wong and Baud (2012) and Heap and Violay (2021) for sedimentary and volcanic rocks, respectively). The compaction bands in the Volvic trachyandesite, however, formed at an angle of ∼10°–30° to the plane perpendicular to the maximum principal stress (Figures 8a and 8b). Although these compaction bands formed at an angle to the maximum principal stress, we did not observe any obvious evidence of shear (Figure 8b).

**Figure 9:**
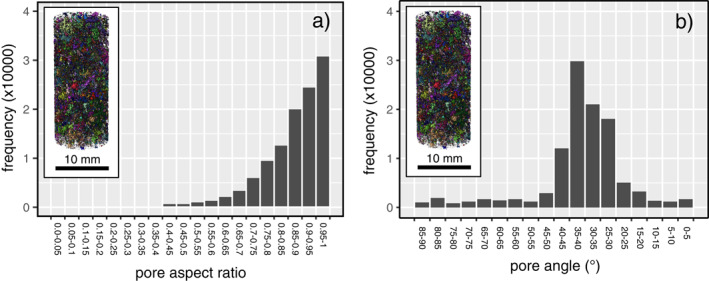
Histograms of (a) pore aspect ratio (the ratio of the minor to the major pore axis, where a value of unity represents a sphere) and (a) pore angle (the angle of pore major axis, where 90° is parallel to the axis of the cylindrical sample) for intact Volvic trachyandesite. This pore shape analysis was performed on the pores not connected to the porosity backbone (i.e., the image volume shown in Figure 2d). The insets show an image of the segmented 3D X‐ray CT volume used for the analysis.

To explore why the compaction bands grew at an angle of ∼10°–30° from the horizontal plane, we performed pore shape analysis on the pores not connected to the porosity backbone (i.e., the image volume shown in Figure 2d). Histograms of the pore aspect ratio (the ratio of the minor to the major pore axis, where a value of unity represents a sphere) and pore angle (the angle of pore major axis, where 90° is parallel to the axis of the cylindrical sample) are given as Figures 9a and 9b, respectively. These data show that, although the majority of the pores are nearly spherical (with an aspect ratio >0.9), there are many pores that have a stretched or elongated shape (e.g., ∼10%–15% of the pores have an aspect ratio <0.7). Figure 9b shows that the major pore axis of the majority of pores is orientated at ∼25°–40°, very similar to the angle of the compaction bands (∼10°–30° from the horizontal plane). We therefore conclude that the compaction bands did not grow perpendicular to the maximum principal stress due to a pore shape preferred orientation. The reason for this is twofold. First, compaction bands can only exist where there are pores, and so the geometry of a compaction band will be influenced by the void space anisotropy. Second, pores are weaker when deformed perpendicular to their major pore axis (Bubeck et al., 2017; Griffiths et al., 2017) and so, for a sample containing elliptical pores, the compaction bands are likely to preferentially form parallel to any major pore axis preferred orientation (presumably as long as this preferred orientation is not parallel or sub‐parallel to the maximum principal stress).

So‐called discrete compaction bands in sandstones are associated with large reductions of several orders of magnitude in permeability (Baud et al., 2012). However, the permeability of our samples deformed at effective pressures of 90 ≤ *P*_e_ ≤ 150 MPa was only reduced by up to an order of magnitude at the maximum axial strain of 0.1–0.12 (Figures 4 and 5), more consistent with the tortuous or diffuse compaction bands described in Baud et al. (2012). Indeed, a detailed microstructural inspection of the compaction bands formed in Volvic trachyandesite confirms that they are far from discrete (Figure 8). The larger reductions in the permeability (by more than an order of magnitude) of other porous volcanic rocks in the ductile regime (Farquharson et al., 2017; Heap, Baud, et al., 2020; Heap et al., 2015) highlights that compaction bands in volcanic rocks can exert a greater influence on permeability than measured here. The influence of a compaction band on the permeability of porous volcanic rock likely depends on the initial microstructural attributes of the rock and, in particular, the spatial distribution of pores (the compaction bands can only exist where there are pores), their connectivity, and their major pore axis preferred orientation. Our data and observations suggest that void space anisotropy in volcanic rocks may greatly influence the geometry of a compaction band and therefore its influence on permeability. More data on a porous volcanic rock with a void space anisotropy, deformed in different directions relative to the major pore axis preferred orientation, are now required to better understand the influence of microstructure on compaction localization and permeability evolution in the ductile regime. We highlight that in‐situ X‐ray CT (e.g., Cartwright‐Taylor et al., 2020; Huang et al., 2019; Renard et al., 2019) and/or the location of AEs (e.g., Brantut, 2018; Fortin et al., 2006; Lockner et al., 1991) during deformation could help provide new insight into the growth of compaction bands in porous volcanic rock.

### 4.4 Quantitative Analysis of Permeability Evolution

We will now investigate relationships between (a) permeability and porosity and (b) permeability and effective mean stress for Volvic trachyandesite, and compare these relationships with those for porous sedimentary rocks.

First, we use a general power‐law description for the porosity‐dependence of permeability *k*(*ϕ*), where *ϕ* is the porosity, *ϕ*_0_ is a reference porosity (here taken to be the initial value), *k*_0_ is a reference permeability (here taken to be the permeability at *ϕ* = *ϕ*_0_), and *n* is the power law exponent:

(2) $$k=k_{0}{\left(\frac{\phi }{\phi_{0}}\right)}^{n}.$$

Similar power‐law approaches are used widely in permeability‐porosity descriptions of volcanic rocks (Klug & Cashman, 1996; Mueller et al., 2005; Wadsworth et al., 2016). While it is desirable to have a theoretical underpinning for choosing *ϕ*_0_ and *k*_0_, here we use these parameters as expedient fit parameters, and focus instead on the value of *n*, the percolation transport exponent, which controls the slope of log(*k*) as a function of log(*ϕ*).

**Figure 10:**
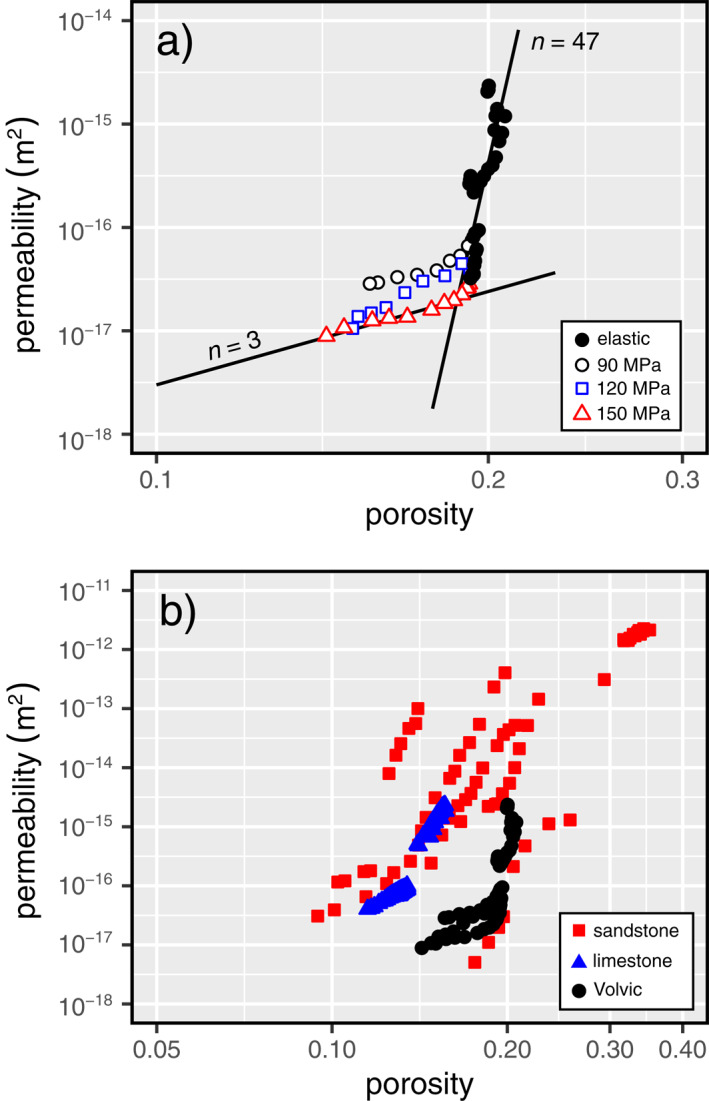
(a) Permeability as a function of porosity for the elastic data (i.e., *Q* < *C*′ and *Q* < *C* ^∗^, and data from the hydrostatic experiment shown in Figure 2) (filled, black circles) and inelastic data for the experiments conducted at effective pressures of 90, 120, and 150 MPa (i.e., *Q* > *C* ^∗^) (unfilled symbols). Black curves are modeled curves for best‐fit values of *n* to the elastic data (*n* = 47) and the inelastic data (*n* = 3), using Equation 2. (b) Permeability as a function of porosity for the elastic and inelastic data in the ductile regime for Volvic trachyandesite during hydrostatic and triaxial deformation experiments (black circles), elastic and inelastic data for porous sandstones during hydrostatic experiments (David et al., 1994) (red squares), and elastic and inelastic data for porous limestones during triaxial deformation experiments (Meng et al., 2019) (blue triangles).

First, we take the data for *k*(*ϕ*) for the samples in the brittle (20 < *P*_e_ < 60 MPa) and ductile (90 < *P*_e_ < 150 MPa) regimes and examine the permeability data for where *Q* < *C*′ and *Q* < *C* ^∗^, data for which the deformation is considered to be elastic. We also consider here data from the experiment in which permeability and porosity were measured during hydrostatic loading to a *P*_e_ of 150 MPa (data also considered to be elastic, since *P*_e_ < *P* ^∗^, where *P* ^∗^ is the *P*_e_ required for inelastic hydrostatic deformation). In this elastic regime, the data *k*(*ϕ*) are steep, and characterized by an apparent *n* that appears largely independent of *P*_e_ and which takes a best‐fit value *n* ≈ 47 (Figure 10a). As discussed above, due to the low pre‐existing microcrack density (Figure 2a), we consider that permeability reductions during pressurization and loading in the elastic regime are the result of the closure or “pinching” of narrow pore throats that help connect the porosity backbone. To give an example for comparison, expansion of overlapping bubble networks is thought to induce a change in porosity and permeability with an *n* value around 2.7 or up to *n* = 3 (e.g., Mueller et al., 2005; Vasseur et al., 2020). Similarly, volcanic welding and sintering changes porosity and permeability coincidently with a value of *n* around 4.2–4.4 (e.g., Wadsworth et al., 2021, 2017). These values are substantially lower than the value for the triaxial compression of the trachyandesite studied here in the elastic regime. However, this *n* value for Volvic trachyandesite in the elastic regime is more consistent with previous work in triaxial compression for sandstones (David et al., 1994; Yale, 1984) and limestones (Meng et al., 2019), for which *n* can be up to 25 in the case of Adamswiller sandstone (Figure 10b). We consider that the higher value of *n* for Volvic trachyandesite is likely the result of its complex pore structure (Figure 1) compared to typical sandstones and limestones, in which the connectivity of the void space likely depends heavily on narrow channels that can be closed or “pinched”.

In the brittle field at *Q* > *C*′, the localized nature of the deformation and damage makes it difficult to quantitatively understand the change in *k*(*ϕ*) except to note that it deviates from the high *n* behavior at *Q* < *C*′ that appears to be independent of *P*_e_. However, in the ductile regime of high *P*_e_ at *Q* > *C* ^∗^ we find that the data for *k*(*ϕ*) deviate smoothly and consistently from the high‐*n* regime found below *C* ^∗^ to a lower‐*n* trend (Figures 10a and 10b). This switch to a lower value of *n* at *Q* > *C* ^∗^ is also seen for porous sandstones, but not porous limestones (Figure 10b). The absence of a reduction in *n* in limestones may be related to their dual porosity microstructure consisting of micro‐ and macro‐pores, as discussed in Meng et al. (2019). For the Volvic trachyandesite, we find that 3.5 < *n* < 4.9 (with the lower bound provided by a fit to *P*_e_ = 150 MPa, and the upper bound provided by a fit to *P*_e_ = 120 MPa, both for the data at *Q* > *C* ^∗^). As *Q* ≫ *C* ^∗^ (equivalently, as *P* ≫ *P*_e_), we see that *n* = 3 provides a reasonable bulk trend to all the data at *P*_e_ > 90 MPa (Figure 10a). For the sandstone data presented in David et al. (1994), we find that 3.8 < *n* < 15.3 for *P* > *P* ^∗^ (i.e., in the inelastic regime). This analysis suggests that as microstructural damage accumulates in the ductile regime, which is localized in the material studied herein (Figures 8a–8c), the data converge toward a *k*(*ϕ*) trend characterized by a low‐*n*. Interestingly, the value *n* = 3 is typical of, and theoretically derived for, granular media such as packed particles (e.g., Carman, 1997; Vasseur et al., 2021; Wadsworth et al., 2021). This leads us to suggest that the damage is atomistic in the sense that the internal structure of the sample is effectively granulating as far as the pore network geometry is concerned. It is interesting to note that cataclastic pore collapse does evolve the microstructure from a groundmass embedded with pores (Figures 1a and 1b) to one that is more granular, as shown by the microstructure presented by the sample deformed to an axial strain of ∼0.11 (Figure 8e).

**Figure 11:**
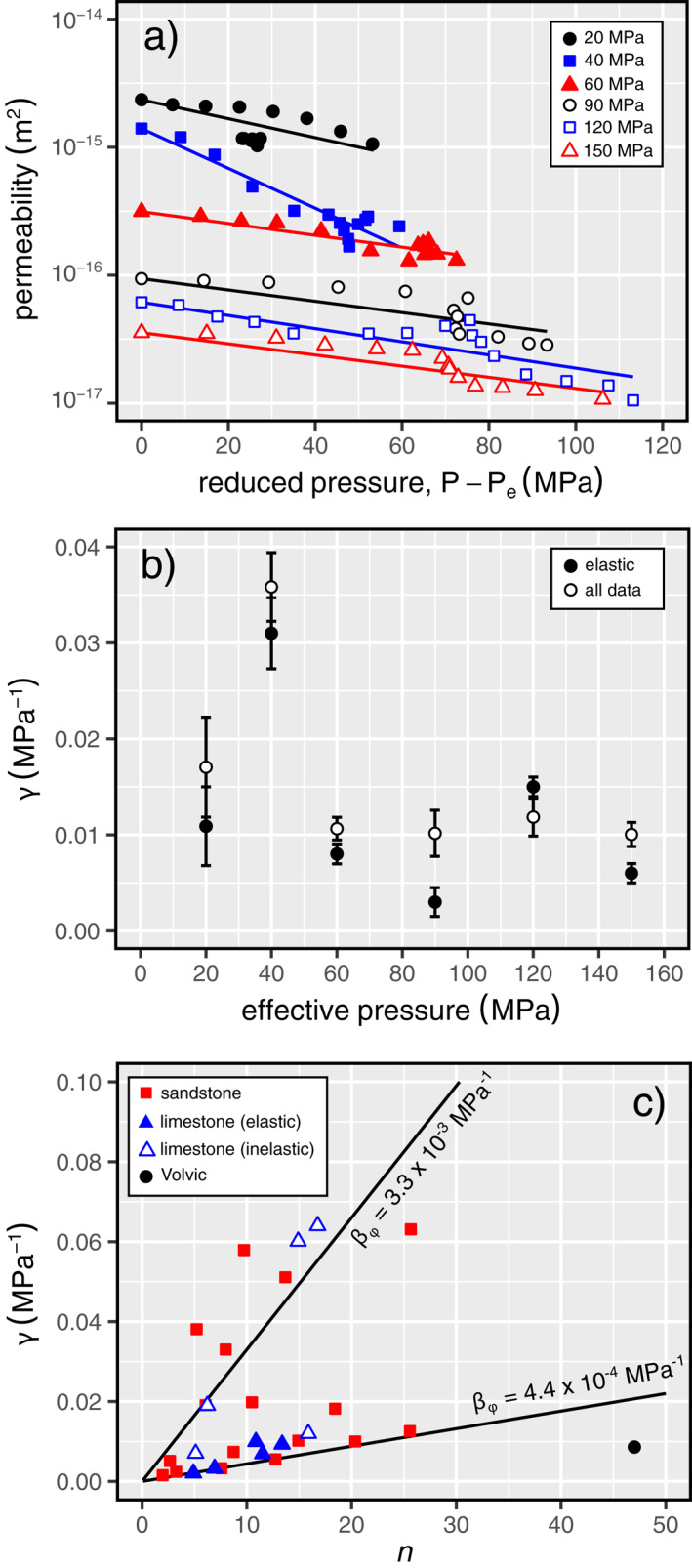
(a) Permeability as a function of reduced pressure, *P* − *P*_e_, along with general fits (all data) for pressure sensitivity coefficient, *γ*. (b) Pressure sensitivity coefficient, *γ*, as a function of effective pressure for the entire data set (elastic and inelastic data) (white circles) and for the elastic data only (black circles). (c) Pressure sensitivity coefficient, *γ*, as a function of porosity sensitive parameter, *n*, for Volvic trachyandesite (black circles) and compiled data for sandstones (red squares; data from David et al., 1994; Yale, 1984) and limestones (blue triangles; data from Meng et al., 2019). Curves are shown for pore compressibilities, *β*_*ϕ*_, of 4.4 × 10^−4^ and 3.3 × 10^−3^ MPa^−1^.

Next, we can investigate the relationship between *k* and *P*. David et al. (1994) suggest an exponential relationship given by:

(3) $$k=\;k_{0}\;\exp\left(-\gamma \left(P-P_{e}\right)\right)$$

where *γ* is a compression coefficient with dimensions of inverse pressure. As with Equation 2, we take *k*_0_ to be an initial value for a given data set (i.e., at *Q* = 0 MPa). Therefore, *γ* is the single unknown in Equation 3. We can fit for *γ* either (a) individually to the whole data set for each *P*_e_, (b) only for data for which elastic deformation was dominant (i.e., for *Q* < *C*′ and *Q* < *C* ^∗^), or (c) only for the data in the ductile regime (*P*_e_ ≥ 90 MPa) at *Q* < *C* ^∗^. In all cases, we find similar results for *γ* such that 0.003 < *γ* < 0.036 MPa^−1^. In Figure 11a we show our data for *k* as a function of *P* − *P*_e_ along with general fits for *γ*, and *γ* is plotted as a function of *P*_e_ in Figure 11b. However, we suggest that Equation 3 is most relevant for purely elastic compression (as noted by David et al., 1994) and so the average *γ* for each *P*_e_ in the elastic regime at *Q* < *C*′ and *Q* < *C* ^∗^, is *γ* = 0.0086 ± 0.0021 MPa^−1^. This value is similar if we follow case (c) mentioned above and only fit these data in the ductile regime of *P*_e_.

If we set Equations 2 and 3 equal to one another, and we assume that $P\to P_{e}$ (such that (*P* − *P*_e_) → 0), we note that Equations 2 and 3 imply a relationship between *γ* and *n* via a bulk compressibility *β*_*ϕ*_ (as noted by David et al., 1994), which is:

(4) $$\gamma =\;-\frac{n}{\phi }\frac{d\phi }{dP}=n\beta_{\phi }.\;$$

We plot on Figure 11c the range of *β*_*ϕ*_ that brackets the data for porous sandstones from David et al. (1994), where *β*_*ϕ*_ = 4.4 × 10^−4^ and 3.3 × 10^−3^ MPa^−1^. Data for tight sandstones (from Yale, 1984), however, plot above this range (Figure 11c). With the exception of the deformed Indiana limestone data, the data for porous limestone from Meng et al. (2019) also fit within these brackets (Figure 11c). Because the derivation of the relationship between *n* and *γ* implies that *P* should be close to *P*_e_, we limit our analysis here to the elastic regime. Therefore, for Volvic trachyandesite, we find values of *n* and *γ* of 47 and 0.0086, respectively. Our new data for Volvic trachyandesite plot below the range of *β*_*ϕ*_ for sandstone delineated by David et al. (1994) (Figure 11c), indicating that the pore space is more compliant than porous sandstones and limestones in the elastic regime. We interpret this compliance as due to the presence of numerous narrow pore throats, not typically present in porous sandstones and limestones, that are important for fluid flow, but can be closed or “pinched” at low pressures. If we were to plot our data in the *γ* *−* *n* plot of Figure 11c for *Q* > *C* ^∗^ (i.e., *n* ≈ 3), then our data would indeed fall in the range of *β*_*ϕ*_ found for sandstones (David et al., 1994), indicating that the compliance of the pore space is lower in the inelastic regime. This reduction in compliance is likely because the porosity reduction during shear‐enhanced compaction is driven by the cataclastic closure of pores (Figure 8), rather than the closure of narrow pore throats.

## 5 Conclusions

In this study, we measured the evolution of porosity and permeability (in the direction of the maximum principal stress) of a porous volcanic rock during deformation in the brittle and ductile regimes. Although data of this type are available for porous sandstones and limestones, the data provided here are a first for porous volcanic rock.

In the brittle regime, the permeability of Volvic trachyandesite decreases by a factor of 2–6 up to the peak stress due to the closure of preexisting microcracks but, following shear fracture formation, remained more‐or‐less constant as strain was accommodated by sliding on the resultant shear fracture (Figures 4 and 5). The absence of permeability changes upon shear fracture formation and during sliding on the fracture can be explained by the fact that the shear fractures did not connect the top and bottom of the samples (Figure 3d). We can conclude, however, that the permeability of the shear fracture did not fall below that of the host‐rock during deformation up to an axial strain of ∼0.08.

In the ductile regime, sample permeability decreased during the initial loading phase, but then continued to decrease, by up to an order of magnitude, as a function of increasing deformation up to an axial strain of ∼0.12 (Figures 4 and 5). Porosity and permeability loss in the ductile regime are driven by the cataclastic collapse of pores (compare Figure 8 with Figure 1a). Compaction in the ductile regime is localized in compaction bands, planes of collapsed pores connected by microcracks (Figures 8b and 8c). Rather than forming perpendicular to the maximum principal stress, the compaction bands formed at an angle of ∼10°–30° to the horizontal plane (Figure 8b), which we show using X‐ray CT to be the result of a pore shape preferred orientation (Figure 9b). The permeability of the sample is not reduced substantially due to the tortuous and diffuse nature of the compaction bands (Figures 8a–8c). Large reductions in permeability resulting from compaction localization are possible when discrete compaction bands form (Baud et al., 2012). However, we do not rule out the possibility of some porous volcanic rocks forming discrete bands, and conclude that microstructural attributes, such as pore size, pore shape, and the spatial distribution of pores, will likely influence the type and geometry of compaction bands in volcanic rocks, and therefore their influence on permeability.

The method of measuring permeability during deformation adopted here has been used previously on porous sandstones (Baud et al., 2012; Fortin et al., 2005; Zhu & Wong, 1997) and porous limestones (Meng et al., 2019), allowing our new data for volcanic rocks to be easily compared with previously published data. We find that the evolution of permeability for porous sandstones and limestones as a function of axial strain is qualitatively similar to that of Volvic trachyandesite: deformation in the brittle and ductile regimes are associated with permeability reductions (Figures 4 and 5). Quantitatively, however, there are differences. For example, the porosity sensitivity exponent of permeability in the elastic regime is higher for Volvic trachyandesite than found previously for porous sandstones and limestones (Figure 10), and is likely the result of the elastic closure or “pinching” of narrow pore throats in Volvic trachyandesite that are important for the connectivity of the void space (Figure 1b). This exponent decreases during shear‐enhanced compaction toward a value typical of, and theoretically derived for, granular media such as packed particles (Figure 10), suggesting that the material is effectively granulating as far as the pore network geometry is concerned. Similar behavior is seen for sandstones in the inelastic regime (at pressures exceeding the grain crushing pressure during hydrostatic experiments; David et al., 1994), but not for limestones (data from Meng et al., 2019) (Figure 10b). Indeed, microstructural observations suggest that cataclastic pore collapse in Volvic trachyandesite evolves the microstructure from a groundmass embedded with pores to one that is more granular (Figure 8e). If the evolution of porosity‐permeability in porous volcanic rocks in the ductile regime approximates that of granular medium, it will simplify future modeling endeavors that require an understanding of how porosity and permeability evolve at depth within a volcanic edifice.

The permeability of volcanic rock controls the distribution of pore fluids and pore fluid pressure within a volcanic edifice. It is therefore considered to exert influence on eruptive style (effusive or explosive; Melnik et al., 2005) and volcano deformation (Farquharson, Heap, Baud, Reuschlé, & Varley, 2016; Heap et al., 2021; Reid, 2004). The lithostatic pressure acting on edifice‐forming rock will increase as volcanic rock is buried by the products of subsequent eruptions, and endogenous stresses are transferred to the host‐rock by, for example, dyke emplacement and magma accumulation (e.g., cryptodome formation). The highly fractured and deformed nature of volcanic structures and the volcanic rocks at active and dissected volcanoes serves as a testament to these pressures and stresses. Our experimental study describes the evolution of porosity and permeability for a porous volcanic rock subjected to elastic deformation and inelastic deformation in both the brittle and ductile regimes (representative of rock deforming in the shallow and deep edifice). These data highlight that deformation throughout the edifice can be expected to influence the distribution of pore fluids and pore fluid pressure within the edifice. Brittle deformation in the shallow edifice (<1 km) could increase or decrease permeability, depending on whether the fault or fracture presents a conduit for or barrier to fluid flow, which could promote or inhibit the outgassing of the magma‐filled conduit (which, in turn, promotes effusive and explosive behavior, respectively; Melnik et al., 2005). Deformation in the deeper edifice (>1 km) can either be brittle or ductile, depending on factors such as porosity (e.g., Heap et al., 2015), and can also therefore promote or inhibit outgassing and influence the style of eruption. Further, brittle deformation capable of reducing permeability and ductile deformation at depth can also promote volcano instability and collapse by increasing pore fluid pressures (Heap et al., 2021; Reid, 2004), and large‐scale volcano spreading, driven by ductile deformation, is also known to jeopardize volcano stability and promote catastrophic collapse (van Wyk de Vries and Francis, 1997). The data of our study can also be used in modeling designed to better understand, for example, fluid flow and pore pressurization within a volcanic edifice (e.g., Chevalier et al., 2017; Collinson & Neuberg, 2012; Collombet, 2009). Alternatively, these data can be used to predict permeability changes in an edifice based on time‐lapse geophysical data, such as muon tomography (e.g., Rosas‐Carbajal et al., 2017; Tanaka et al., 2010). Ultimately, a robust understanding of the evolution of porosity and permeability in volcanic rock as a function of elastic and inelastic strain accumulation in the brittle and ductile regimes will improve the accuracy of models designed to assist volcano monitoring and volcanic hazard mitigation.

## Supporting information

Supporting Information S1

Data Set S1

## Data Availability

The data collected for this study can be found in the accompanying Microsoft Excel© spreadsheet. The data can also be downloaded here: https://doi.org/10.6084/m9.figshare.19949474.v1.
