# Supplementary material for: The Permeability of Porous Volcanic Rock Through the Brittle‐Ductile Transition
Source: J Geophys Res Solid Earth. 2022 Jun 20;127(6):e2022JB024600. doi: 10.1029/2022JB024600 (PMC9286468; doi:10.1029/2022JB024600)
Supplement: Supplementary file 1 — Supporting Information S1 [file JGRB-127-0-s001.docx]

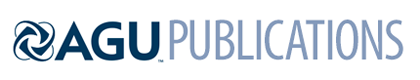


*Journal of Geophysical Research: Solid Earth*

Supporting Information for

**The permeability of porous volcanic rock through the brittle-ductile transition**

Michael J. Heap^1,2,3^, Gabriel G. Meyer^3^, Corentin Noël^3,4^, Fabian B. Wadsworth^5^, Patrick Baud^1^, and Marie Violay^3^

^1^Université de Strasbourg, CNRS, Institut Terre et Environnement de Strasbourg, UMR 7063, 5 rue Descartes, Strasbourg F-67084, France

^2^Institut Universitaire de France (IUF), Paris, France

^3^Laboratory of Experimental Rock Mechanics, Ecole Polytechnique Fédérale de Lausanne, Lausanne, Switzerland

^4^Dipartimento di Scienze della Terra, La Sapienza Università di Roma, Piazzale Aldo Moro 5, 00185 Rome, Italy

^5^Earth Sciences, Durham University, Durham, DH1 4LY, U.K.

**Contents of this file**

Description of Data Set S1

**Additional Supporting Information (Files uploaded separately)**

Description of Data Set S1

**Introduction**

The Supporting Information that accompanies this manuscript is a Microsoft Excel spreadsheet containing all the data collected for this study (Data Set S1).

Data Set S1. All the mechanical and permeability data collected for the study “The permeability of porous volcanic rock through the brittle-ductile transition”.
